# JULIDE: A Software Tool for 3D Reconstruction and Statistical Analysis of Autoradiographic Mouse Brain Sections

**DOI:** 10.1371/journal.pone.0014094

**Published:** 2010-11-23

**Authors:** Delphine Ribes, Julia Parafita, Rémi Charrier, Fulvio Magara, Pierre J. Magistretti, Jean-Philippe Thiran

**Affiliations:** 1 Signal Processing Laboratory (LTS5), Ecole Polytechnique Fédérale de Lausanne (EPFL), Lausanne, Switzerland; 2 Brain Mind Institute, Ecole Polytechnique Fédérale de Lausanne (EPFL), Lausanne, Switzerland; 3 Department of Psychiatry, Centre de Neurosciences Psychiatriques, CHUV and University of Lausanne, Lausanne, Switzerland; University of Queensland, Australia

## Abstract

In this article we introduce JULIDE, a software toolkit developed to perform the 3D reconstruction, intensity normalization, volume standardization by 3D image registration and voxel-wise statistical analysis of autoradiographs of mouse brain sections. This software tool has been developed in the open-source ITK software framework and is freely available under a GPL license. The article presents the complete image processing chain from raw data acquisition to 3D statistical group analysis. Results of the group comparison in the context of a study on spatial learning are shown as an illustration of the data that can be obtained with this tool.

## Introduction

Functional brain imaging techniques such as Positron Emission Tomography (PET) and Magnetic Resonance (MR) have revolutionized the study of brain function in humans. Like for other aspects of neurobiological research, studies in laboratory animals are still indispensable in order to advance our knowledge of basic mechanisms. Small animal imaging devices including PET and MR have been developed over the years [1], [2]. These imaging techniques have been particularly useful to monitor functional activation during specific behavioral modalities as well as in response to pharmacological agents, in particular using ^14^C-2-deoxyglucose uptake as an indirect marker of neuronal activity [3], [4], [5] Although these techniques have great advantages such as the possibility to perform longitudinal studies in the same animal, technical limitations still exist, including low spatial resolution, limited sensitivity and high cost.

Since its introduction by Sokoloff and colleagues (1977), high-resolution autoradiography has been used for several years now to map brain activation; it remains a valid approach and complement to the above-mentioned image modalities, because of its low cost and wide availability. It is the reference technique most widely used in neuroimaging for small animal studies. However a considerable limitation of this technique is the fact that, unlike PET or MR, longitudinal studies in the same animal cannot be performed. Consequently, large numbers of sections from different animals have to be processed, raising a major technical problem, namely the loss of three-dimensional (3D) spatial consistency across brains.

Some of the limitations of high-resolution autoradiography can be overcome by developing automated 3-D reconstruction procedures from large series of sections from the same brain [6], [7], [8], [9], [10], [11] and by then allowing comparisons of radioactivity distribution across regions between animals belonging to the control or experimental group. The latter approach entails the identification of pre-defined regions of interest (ROI), a procedure that can be seriously biased by the observer. More importantly, ROI selection is by definition based on a priori hypotheses, thus introducing considerable subjectivity in the analytical procedure. Procedures that avoid analysis with such prior assumptions have been developed for PET [12] and fMRI [13] human studies. They are based on voxel-wise statistical analysis for comparisons between groups. This approach entails the establishment of a standardized brain volume following spatial normalization of individual brains. This kind of approach has successfully been used in a few behavioral studies in rodents [14], [15], [16]. Furthermore, a fully automated analysis method for autoradiographic data from rodent brains has been recently proposed by Dubois et al [17]. However the adoption and extensive use of those techniques, which involve a significant number of sophisticated methodological steps, is still largely limited by the availability of usable, freely available tools gathering the specific techniques into a user-friendly environment.

In this article, we present a new software tool, called JULIDE, that we developed to perform the 3D reconstruction, intensity normalization, volume standardization by 3D image registration and voxel-wise statistical analysis of autoradiographs of mice. This software tool has been developed in the open-source ITK software framework [18] and is freely available under a GPL license. Results of the group comparison in the context of a study on spatial learning are shown as an illustration of the data that we can obtain with this tool. To the best of our knowledge JULIDE is the only open source and freely available software in this domain.

The article is organized as follows. In the next section, we first describe the different building blocks of JULIDE, ranging from image intensity normalization of brain section images, and their subsequent alignment for 3D reconstruction, to inter-subject 3D registration and voxel-wise statistical analysis. Some implementation details and software availability are also presented at the end of this section. Then an experimental protocol, related to a spatial learning task study, is described at the beginning of the “Results and discussion” section. Results of the group study using this protocol are shown and discussed in that section, where conclusions are also drawn.

## Methods

### Overview

JULIDE is implemented as a series of successive steps that cover the whole processing chain from individual 2D slice images to the 3D statistical maps showing the differences between two groups of subjects. The graphical user interface (GUI) has been designed to allow a simple navigation within this chain, to save intermediate results, reload them for further analysis, etc. It includes both the processing and the visualization blocks to finally display the statistical analysis results.

### Data acquisition, pre-processing and intensity normalization

Cerebral glucose metabolism (CMRGlu) was measured using the [^14^C]-2-deoxyglucose autoradiographic method [19], in adult C57BL/6 male mice, under radial arm maze (RAM) training. A detailed description of the experimental protocol is provided in the next section.

Brain sections (150 per animal, 20 µm thick) were cut with a cryostat at −20 °C, mounted on SuperFrost glass slides, rapidly heat-dried, and exposed to an autoradiographic film for 15 days, together with radioactive [^14^C] standards. The sectioning operation was performed according to stereotaxic coordinates of the Hof et al. mouse brain atlas [20]: each brain was sectioned at 20-µm thickness between bregma 2.5 mm and bregma −4.6 mm in the coronal plane. We used a single 18×24 cm autoradiography film with the proper cassette per mouse brain, the sections being distributed on the film as follows: 2 columns of 9 glass slides and 1 column of 3 glass slides, each one containing 8 sections, and 1 slide bearing [^14^C] standards within the third column. Hence, for each mouse brain, both [^14^C] standards and background were identical for all sections and consequently a single calibration was necessary.

**Figure 1:**
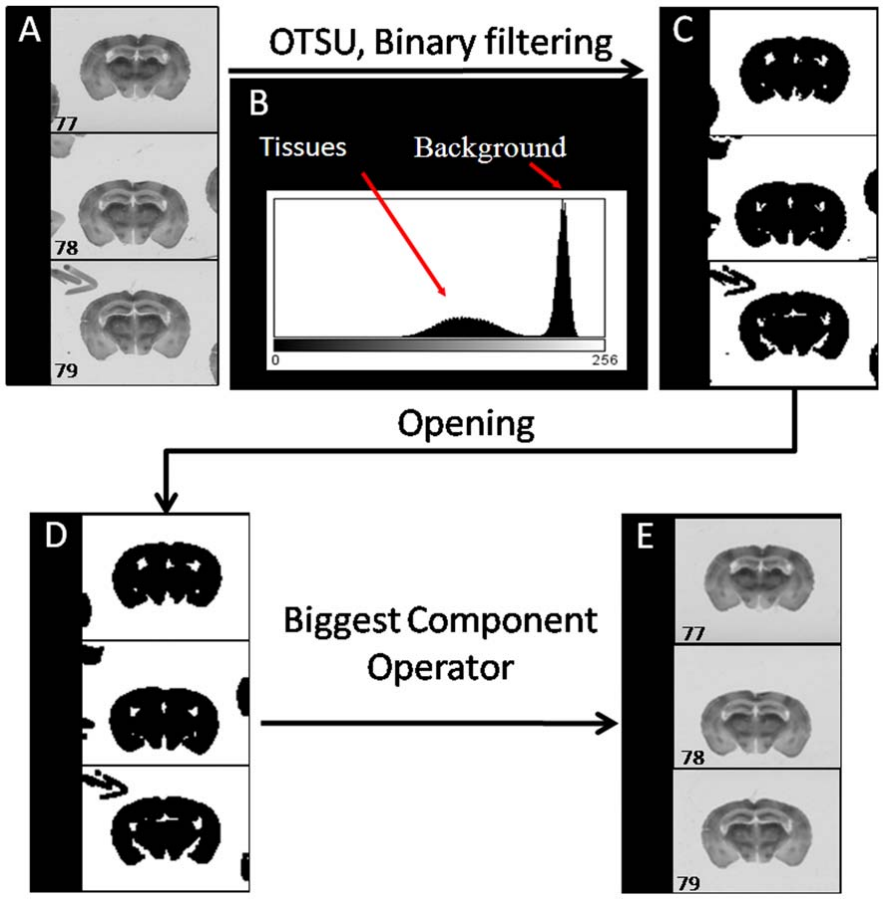
Preprocessing procedure. (A) Sections are stored as individual 2D slices. (B) Histogram of one section. Two modes: Tissues and Background. (C) Binary images resulting from the threshold calculated using OTSU technique. (D) Results of opening operator to clean noise or separate overlapping sections. (E) Individual sections ready for 3D reconstruction.

The autoradiographs are digitized as 8-bit gray-scale images with the MCID software (Image Analysis Software Solutions for Life Sciences, Interfocus Imaging Ltd., Linton, UK). In-plane digitization resolution is chosen sufficiently high to capture fine structures of interest: the pixel size has been set to 10×10 microns. Individual autoradiographic sections are finally manually outlined and stored as individual sections (see figure 1.A) using tiff format.

The gray level intensities obtained from the autoradiographic volume images were calibrated using the co-exposed [^14^C] standard scale, converted to radioactivity values (nCi/g of tissue) using a cubic spline interpolation.

To automatically select brain tissues and exclude artifacts such as overlapping sections or dust, a cleaning procedure is used. It is based on robust histogram analysis and mathematical morphology operators: first, extraction of the two main classes present in the scans, corresponding to the brain tissues and the background, is done via OTSU technique [21]. This technique automatically calculates the optimal threshold *t* to binaries scans. An opening procedure is then applied to separate overlapping sections. Finally only brain sections are kept using the largest connected component in the image (see figure 1).

### Intra-subject 3D reconstruction

**Figure 2:**
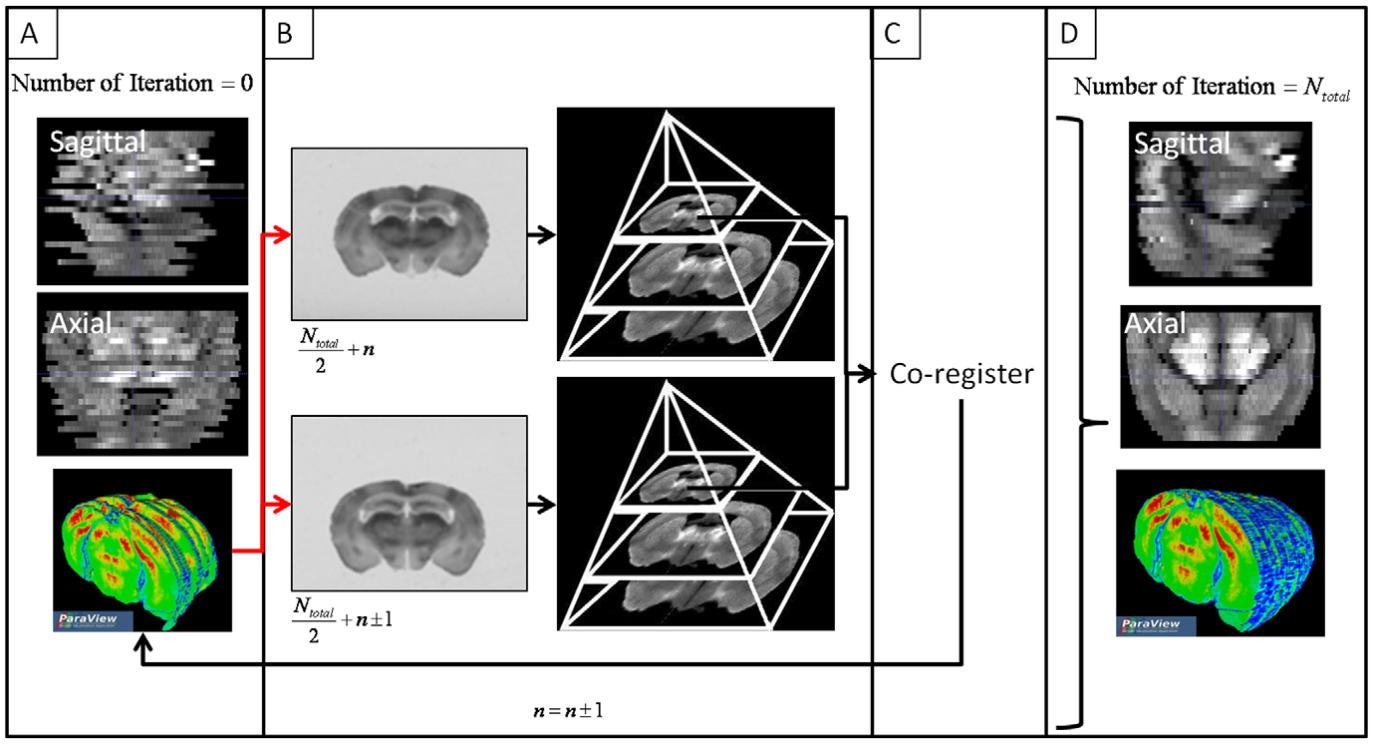
Intra-subject 3D reconstruction procedure. (A) Sections are first stacks in the Z direction. (B) Consecutive sections are extracted and down-sampled by a factor of four (for the first iteration, the two central sections are extracted). (C) Consecutive sections are co-registered and replace in the stacked volume. (D) The procedure ends when all sections have been aligned to the consecutive or the previous one.

First, autoradiographic reconstructed volumes are obtained by stacking coronal sections in the z direction. Each section is then sequentially aligned to the adjacent one, starting with central sections [11]. The alignment is done using a rigid registration (6-degrees of freedom) based on minimizing normalized cross correlation cost function. To reduce registration computation time, each section is first down-sampled by a scaling factor of four (see figure 2A).

### Inter-subject normalization by 3D registration

To perform voxel-based statistical analysis, image data volume need to be normalized into a standardized 3D coordinate space. An image of a normal mouse brain was selected as the reference template. This choice was based on the image quality (no preparation artifacts), degree of symmetry and quality of alignment. In the current approach with one image chosen as reference atlas, we acknowledge that some bias might be induced. There are different strategies for template selection/construction that could be adopted in the future to limit this bias [22]. A first straightforward approach could be to create a template as in [23] where all images in a data set are registered to a reference and an average model is created by applying all the averaged transformations to the averaged deformed images. This leads to an averaged model for the intensity and shape of the object. Instead of simply ‘averaging’, other approaches like STAPLE allow to create a non-bias template [24] in a way that estimated weights are included in the average for every deformed image. Further, group-wise registration methods have been recently proposed to create brain template in humans [25].

**Figure 3:**
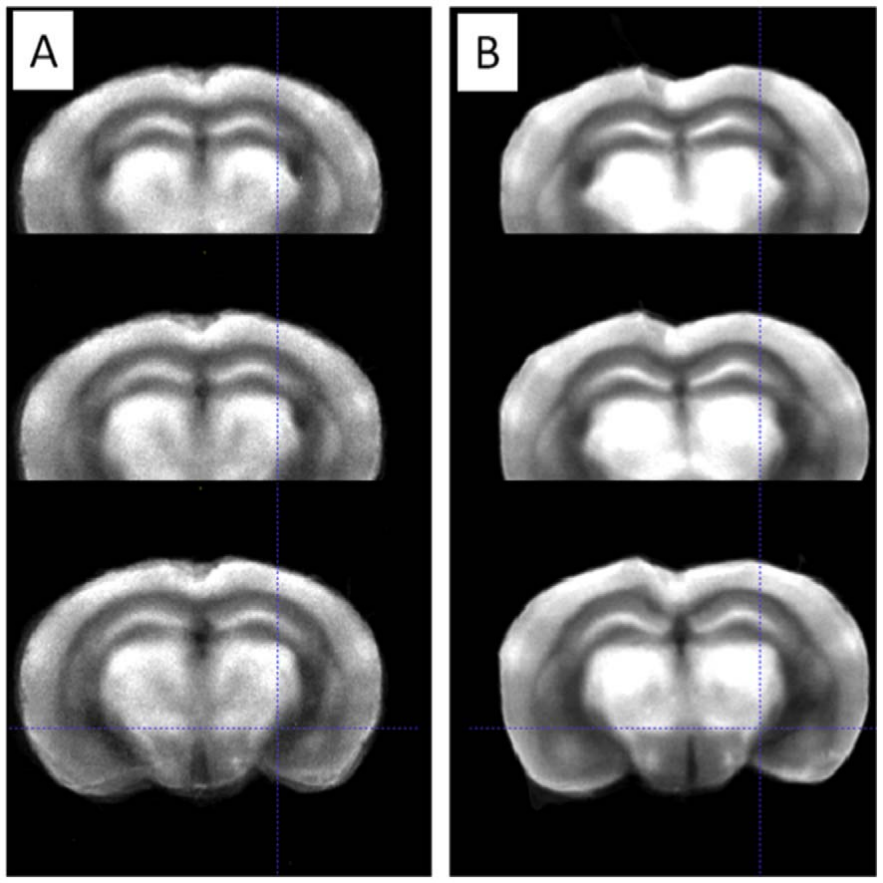
Inter-subject normalization by 3D registration. (A) Average image of a group of 8 mice brain after 3D affine registration. (B) Average image of the same group after 3D non-rigid registration.

In the current implementation, the reference image was first smoothed with a Gaussian kernel (FWHM  = 3 times the voxel size)[17]. To warp each mice brain into the smoothed reference brain, two registrations procedure are used, following the classical practice [13], [26]. First, an affine transformation (12-degrees of freedom) between the image to be normalized and the template is calculated to spatially remove rotation, translation and scaling differences between reconstructed volumes. Then a non-linear transformation (B-spline) is calculated to remove local differences between individuals. Both spatial normalizations are carried out using the ITK multi-resolution scheme to reduce computation time and use normalized cross correlation cost function [18], [22]. Figure 3 illustrates the difference in alignment quality before and after non-rigid registration. The importance of such a non-rigid registration technique is clearly visible in that figure, where important regions such as the cortex, the hippocampus or the hypothalamus are clearly better aligned with non-rigid registration than with a simple affine registration, as reflected by the better sharpness of the image in those regions.

#### Statistical analysis

The spatially normalized images are smoothed using a Gaussian kernel of three times the voxel dimension to increase signal-to-noise ratio and to account for variations in the subtle anatomical structures. Statistical comparison between groups can be made with JULIDE: a voxel-wise student t-test is performed between the data of two groups, to identify the regions with significantly different activation. Maps of positive and negative *p* value, corresponding to voxels for which activations are higher or lower, respectively, in control or activated brain can be analyzed separately. Controlling for the Family wise Error Rate (FWE) and False Discovery Rate (FDR) due to multiple testing has been introduced.

**Figure 4:**
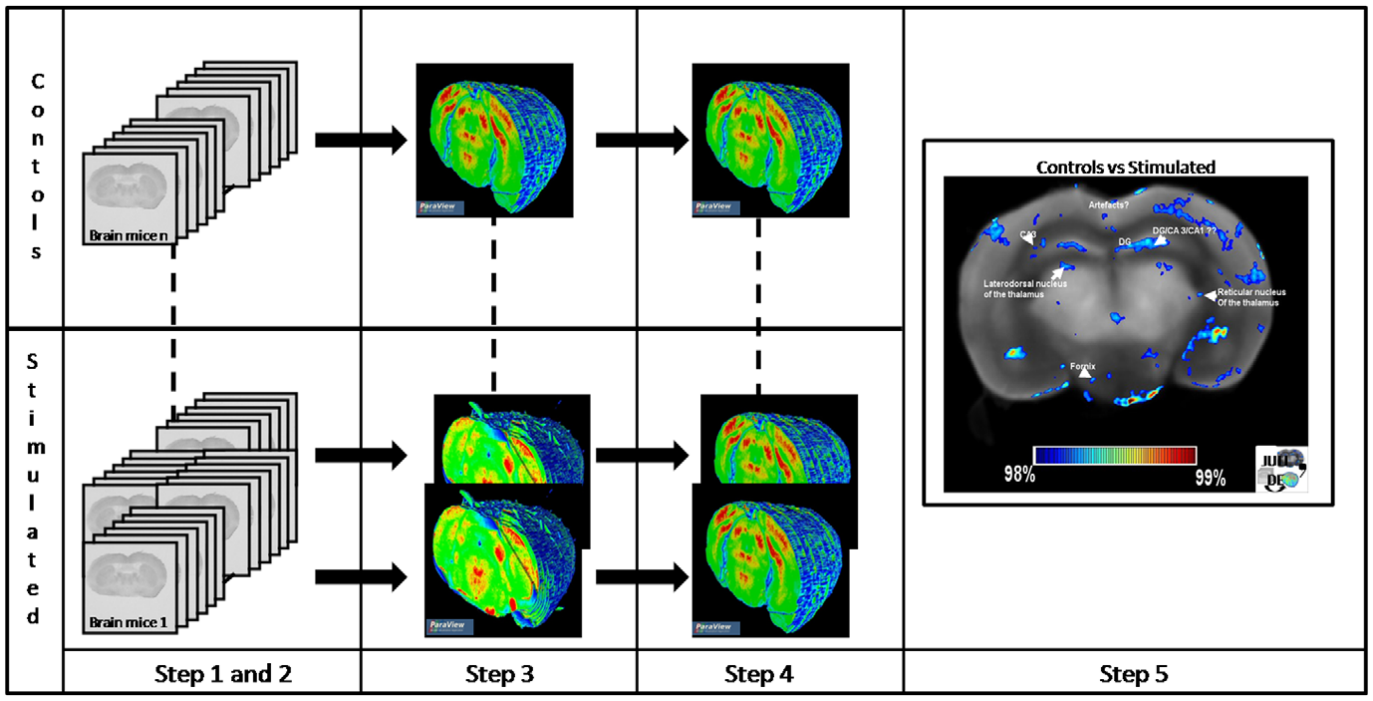
JULIDE framework. JULIDE is a 5 steps processing. Step 1. Pre-Processing: Cleaning of the autoradiographs from dust or overlapping sections. Step 2. Calibration: grayscale intensities are linearly calibrated using the co-exposed [14C] standard scale. Step 3. 3D reconstruction. Step 4. Spatial normalization to the chosen reference space. Step 5. Statistical analysis and results visualization.

This whole 5-step process is illustrated in figure 4.

### Implementation

All computerized treatments and procedures presented in this article (preprocessing, intensity normalization, 3D reconstruction, spatial normalization and statistical analysis) are written in C++ using ITK libraries for image processing [18] and FLTK for GUI [27].

The GUI of JULIDE permits to navigate through the steps, from pre-processing of the autoradiographs to the statistical group analysis. The software can be seen as a five step processing chain. Each step can be performed sequentially or in a row. The first step consists in automatically cleaning the autoradiograph from dust or overlapping sections. The second step performs the calibration of the gray level of the autoradiograph using the co-exposed [^14^C] standard scale. It also stacks coronal sections into the z direction. The third step performs intra-subject 3D reconstruction. A number of volumes can be selected at the same time to perform reconstruction. The fourth step does the spatial normalization. The mouse brain sections used as reference space must be first selected and reconstructed. Then when each mouse brain to analyze is spatially transformed into the reference space, the statistical analysis can be performed. It consists in clustering spatially normalized brains into control or stimulated brain and applying t-test to those groups. Maps of positive and negative p value, corresponding to voxels for which activations are higher or lower, respectively, in control or stimulated brain are saved and can be visualized using the GUI. The level of significance and cluster size can be chosen by the user.

JULIDE has been developed as an open-source toolkit, under a GPL license. More details on how to download and how to use it are available at the following URL: http://julide.epfl.ch.

## Results and Discussion

### Experimental protocol: metabolic mapping of a spatial memory trace formation

Animals (C57BL/6 male mice from Janvier, France) were trained on a spatial learning paradigm, the eight-arm radial maze (RAM). The RAM was introduced by David Olton and Robert Samuelson in 1976 [28]. The original apparatus was designed with eight radial arms and was thought to study spatial learning in rats. Later, the same device but adapted for mice was developed [29]. The number of arms can differ enormously from one study to another, researchers using maze paradigms ranging from 3 to 48 arms [30].

The learning task consisted of correctly find 3 specific arms in the maze of 8 arms with the help of spatial cues on the walls of the room. These 3 arms were baited with ∼10 µl of condensed milk diluted 1∶1 with water, which is highly motivating for mice to visit and explore the device. Spatial learning was assessed by calculating the percentage of entries in correct arms (the 3 arms baited) out of the total number of entries. Animals were trained during 9 days, doing 6 trials per day.

In relation to this training task, we wanted to estimate the level of functional activity in brain regions by measuring the energy metabolism (glucose consumption) using a radioactive analogue of glucose, the ^14^C-2 Deoxy-D-Glucose (2DG)[19]. Glucose consumption was measured during and after the learning task and also at two different times during the training protocol, Day 1 and Day 9. Two different control groups were added to the experiment to ensure that the brain metabolic activation we observed was caused by the spatial learning process. The first one was the Quiet Control (QC), animals that never go to the RAM. The second one was the Active Control (AC), animals that were allowed to explore the maze during the same time as trained animals (1 or 9 days, 6 trials/day) but with all eight arms repeatedly baited with food, thus avoiding a cognitive demand.

The 2DG technique has its advantages (as the individual brain analysis for each animal allowing us to study the correlation between good or bad individual performance and a given pattern of brain metabolic activation), but one inconvenient aspect is the biased analysis one does and the subsequent (possible) omission of some, maybe, important activated regions. One must also consider that this type of analysis is time-consuming.

That was the reason why we decided to develop a new software to make the analysis of autoradiograms (2DG brain sections) in a faster, more efficient and unbiased manner.

Region-of-Interest (ROI)-based analysis of autoradiograms before developing JULIDE has been done using the MCID software (Image Analysis Software Solutions for Life Sciences, Interfocus Imaging Ltd., Linton, UK). After brain slices exposure to autoradiographic films during 15 days, as described in the “Methods” section, films were developed and brain sections stained with cresyl violet. Images of both, stained and autoradiographic sections, were taken using MCID. Stained brain sections were used to identify and delineate the ROIs while optical densities were determined on the corresponding autoradiograms, since both types of images were superimposed. Optical densities were converted to glucose consumption values, as 2DG uptake in nCi/g, based on the co-exposed ^14^C standard scale.

### Analysis of the results and discussions

We have applied this mapping approach JULIDE to the study of the regional variations in CMRGlu in mice undergoing a spatial learning task. Significant differences in the areas engaged during the behavioral task at day 1 (when animals are confronted for the first time to the maze) and at day 9 (when animals are highly performing) have been identified. These areas include the hippocampus, the parietal cortex, the anterior cingulated and the retrosplenial cortex.

**Figure 5:**
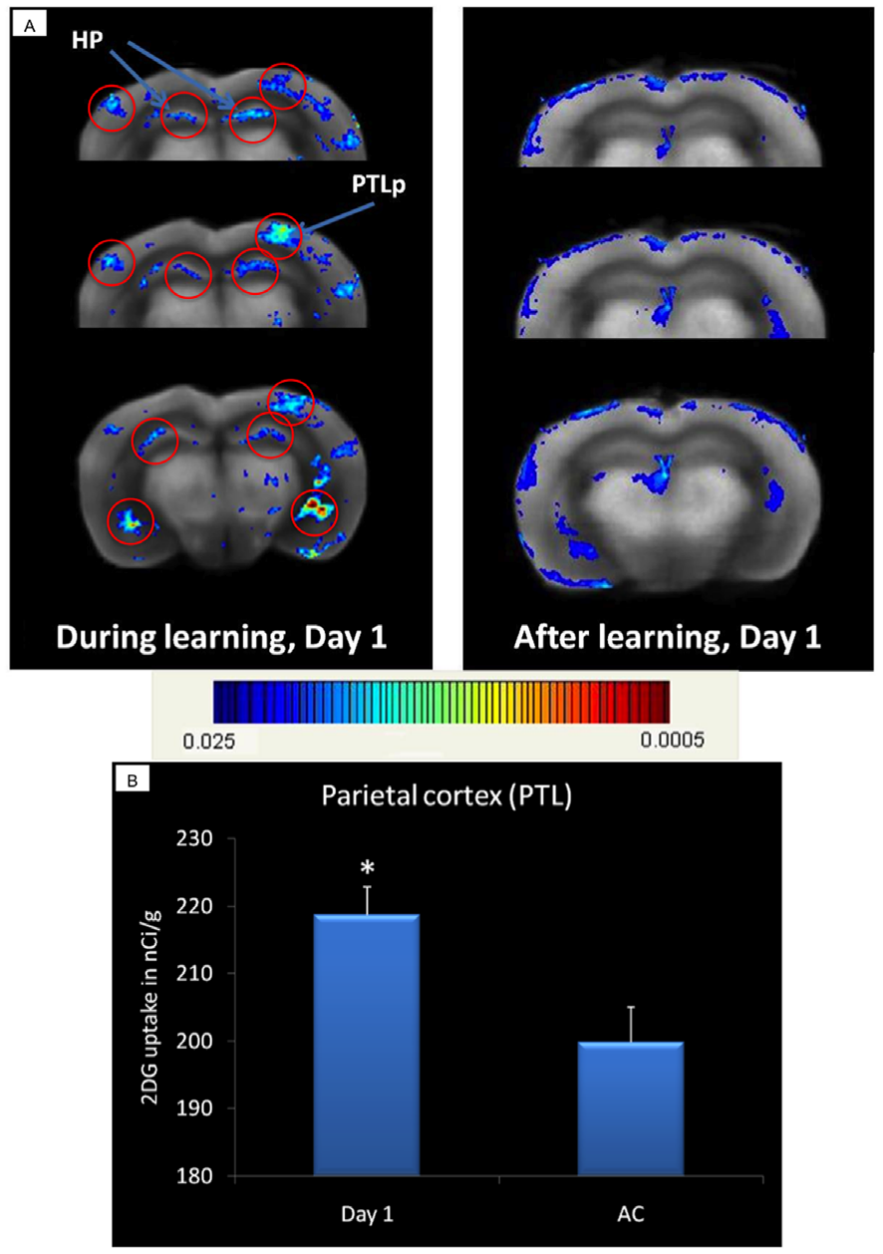
Activation results during learning (Day 1). A: JULIDE results. At Day 1, the hippocampus (HP) and the parietal associative cortex (PTLp) are the most activated regions during learning, whereas after the task they are no longer activated. t-test comparison between Day 1 trained mice and Active Control (AC). Uncorrected p-value  = 0.02. Clusters circled in red show significant activation after FWE and FDR corrections (corrected p-values <0.01). B: MCID results for the parietal associative cortex (PTLp). Increase in glucose consumption, measured as 2DG uptake, in the parietal cortex (PTL) the first (Day 1) day of training. t-test comparison between Day 1 trained mice (n = 7) and Active Control (AC; n = 8). *p-value <0.01.

Figure 5A shows the activation map obtained with JULIDE during the first day of training. Clusters circled in red are the significantly activated ones (p-value lower than 1% after FWE and FDR correction. As we can see, the hippocampus, an area that is well known to be implicated in spatial memory, is activated during the learning task the first day of training. The other activated region we can see on this figure is the parietal associative cortex that integrates sensory information from different modalities as vision, touch and audition, being involved in spatial navigation and visual processing. The t-test comparison was done between trained mice for one day and Active Controls. In figure 5B, results obtained from the MCID software concerning the parietal associative cortex show an increase in glucose utilization (about 10% more) the first day of training (Day 1) when compared to the Active Control, which is corresponding to JULIDE results showing in figure 5A. Notice that a strong activation appears in the ventricles, which obviously reveals variability in periventricular shape across animals, preventing a perfect alignment of the datasets in this region.

**Figure 6:**
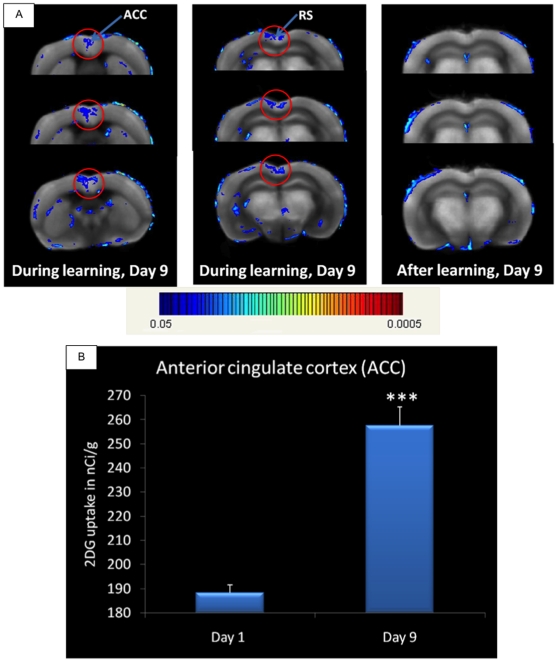
Activation results during learning (Day9). A: JULIDE results. Activation of the anterior cingulate (ACC) and the retrosplenial cortex (RS) during the last day of training, Day 9. No activation of the hippocampal formation is observed, meaning that the recruitment of 2DG has moved, over time, from the hippocampus to the cortex. Images of the post-learning condition are also included in the picture to show that no activation was found after the end of the task. t-test comparison between Day 9 and Day 1 trained mice. Uncorrected p-value  = 0.05. Clusters circled in red show significant activation after FWE and FDR corrections (corrected p-values <0.01). B: MCID results for anterior cingulate cortex (ACC). Glucose consumption, measured as 2DG uptake, in the anterior cingulate cortex (ACC) during the first (Day 1) and the last (Day 9) day of training. t-test comparison between Day 9 (n = 7) and Day 1 (n = 7) trained mice. ***p-value <0.001.

At day 9, the last day of training, the hippocampus is no longer activated but a high signal is detected in the anterior cingulate (ACC) and the retrosplenial (RC) cortex (see figure 6A), meaning that the 2DG recruitment has moved, over the nine days of training, from the regions that were activated at day 1 to these cortical areas, which is consistent with theories of memory consolidation [31], [32]. The t-test comparison was done between animals trained one and nine days. Regions circled in red are significantly different after FWE and FDR correction. If we measure glucose consumption by the “classical” optical densitometry system and if we compare the same groups as in figure 6A, Day 1 and Day 9 trained mice, a high increase (37%) can be found in the anterior cingulate cortex the last day of training (Day 9) (see figure 6B), confirming the previous results obtained with the JULIDE software.

Thus we have a rather complete picture of the patterns of regional activations which are differentially engaged at day 1 and day 9. Results obtained with JULIDE are comparable to those obtained manually by optical densitometry of pre-selected ROIs, namely the hippocampus and the retrosplenial cortex [33], the parietal associative cortex (figure 5) and the anterior cingulate cortex (figure 6).

The ^14^C-2 Deoxy-D-Glucose (2DG) autoradiographic technique affords a valuable means to assess in laboratory animals the engagement of brain regions and circuits in a given behavioral task or following a pharmacological intervention, by determining glucose utilization (CMRGlu) associated with neuronal activity. One of the drawbacks of the technique is that it implies an a priori definition of regions of interest (ROI); in addition 3D reconstructions of the whole brain with unbiased identification of activated (or deactivated) regions would be highly desirable, as currently achieved in human functional imaging studies. Recent attempts in this direction have been proposed [15], [17]. The new tool presented in this article achieves such goals, based on merging and warping of brain autoradiograms prepared from mice belonging to the same experimental group, and aimed at the unbiased identification of regions of interest combined with 3D reconstruction.

Software tools such as JULIDE, developed as an open-source tool and freely available, and other recently proposed [15], [17], [34] are likely to provide useful tools to exploit to its full extent the use of autoradiography to complement behavioral and pharmacological studies in laboratory animals.
